# The Utility of Plasma Metanephrines to Optimise Adrenal Vein Sampling for Primary Aldosteronism: A Single Centre Experience

**DOI:** 10.1111/cen.15277

**Published:** 2025-05-19

**Authors:** Zin Htut, Ali Alsafi, Aditi Sharma, Daniel Foran, Tricia Tan, Sophie C. Barnes, Emma L. Williams, Debbie Papadopoulou, Aimee Dimarco, Fausto Palazzo, Jeannie Todd, Rami Fikri, John Wass, Karim Meeran, Florian Wernig

**Affiliations:** ^1^ Division of Diabetes Endocrinology and Metabolism, Imperial College London London UK; ^2^ Imaging Department Hammersmith Hospital, Imperial College Healthcare NHS Trust London UK; ^3^ Department of Surgery & Cancer Imperial College London UK; ^4^ Department of Endocrinology Imperial College Healthcare NHS Trust London UK; ^5^ Radcliffe Department of Medicine University of Oxford Oxford UK; ^6^ Department of Clinical Biochemistry North West London Pathology London UK; ^7^ Department of Endocrine Surgery Imperial College Healthcare NHS Trust London UK; ^8^ Department of Endocrinology University Hospitals Bristol NHS Foundation Trust Bristol UK; ^9^ Oxford University Hospitals Oxford UK

**Keywords:** adrenal vein sampling, cannulation success, lateralisation, mild autonomous cortisol secretion, plasma metanephrines

## Abstract

**Background:**

Primary aldosteronism (PA) is a prevalent yet frequently underdiagnosed cause of secondary hypertension, affecting up to 10% of hypertensive individuals and contributing to increased cardiovascular risk. Accurate diagnosis is vital, as unilateral PA cases typically require surgical intervention, while bilateral disease is managed medically. Adrenal vein sampling (AVS) remains the gold standard for diagnosing subtypes of PA; however, the use of cortisol to confirm accuracy of cannulation poses challenges due to its long half‐life and potential cortisol co‐secretion by aldosterone‐producing adenomas.

**Objective:**

This study evaluates the diagnostic utility of plasma metanephrines (MN) as an alternative to cortisol in assessing cannulation success and lateralisation of aldosterone secretion.

**Methods:**

Analysing 132 unstimulated AVS procedures performed by a single operator on 129 patients with confirmed PA, we established optimal cut‐off values for the selectivity index (SI) and lateralisation index (LI) using MN.

**Results:**

A MN SI cut‐off of >3 achieved 99% sensitivity and 100% specificity, while an aldosterone/MN LI of >4 indicated unilateral disease with 94% sensitivity and 96% specificity.

**Conclusion:**

Our findings demonstrate that incorporating MN measurements significantly enhances the accuracy of AVS interpretations, particularly in cases of cortisol co‐secretion, thereby minimising diagnostic errors and optimising treatment strategies. This study supports the use of MN as reliable analytes to improve the diagnostic accuracy of AVS.

## 1 Introduction

Primary aldosteronism (PA) is a common but often underrecognised cause of secondary hypertension [1]. Recent studies suggest that it may affect up to 10% of individuals with hypertension, leading to challenges such as resistant hypertension and increased cardiovascular risks [2]. Clinical subtypes include unilateral and bilateral disease, with unilateral cases typically treated surgically. Due to the limitations of CT or MRI in reliably distinguishing between these subtypes, adrenal vein sampling (AVS) is essential for precise diagnosis and tailored intervention in patients with confirmed PA [3].

Confirmation of successful cannulation relies on comparing cortisol levels in the adrenal vein (AV) samples to those from the inferior vena cava (IVC) or a peripheral vein (PV). However, cortisol's variable secretion pattern, long half‐life and potential cortisol co‐secretion by aldosterone‐secreting adrenal adenomas can lead to misinterpretation [4]. Cortisol‐co‐secretion is common. In a German cohort of PA patients, 77.6% had a positive result for at least one of three screening tests for autonomous cortisol secretion or Cushing's syndrome: the 1 mg overnight dexamethasone suppression test (ONDST), late night salivary cortisol and/or 24‐h urinary free cortisol [5]. Compared with cortisol, plasma metanephrines (MN) have a short circulating half‐life of 3−6 min, resulting in a nearly 90‐fold increase in AV compared with PV concentrations when catheters are correctly positioned [6, 7]. This substantial gradient offers a potentially more accurate approach for confirming correct catheter placement than smaller gradients observed with plasma cortisol. MN and normetanephrine (NMN) are metabolites of the catecholamines, adrenaline and noradrenaline, respectively, while 3‐methoxytyramine (3‐MT) is the major metabolite of dopamine. These metabolites are produced by the enzyme catecholamine O‐metheyltransferase (COMT) within adrenal chromaffin cells in the adrenal medulla. They are produced continuously within those cells, independent of catecholamine release making them a more reliable analyte than cortisol.

This study aims to assess the diagnostic utility of plasma MN in assessing AV cannulation success and determining lateralisation of aldosterone secretion during AVS. Cut‐off values for selectivity index (SI) and lateralisation index (LI) were established to differentiate between unilateral and bilateral subtypes of PA.

## 2 Materials and Methods

This retrospective study included 131 unstimulated, sequential AVS procedures performed by a single operator (AA) at a tertiary referral centre between January 2018 and May 2023. All patients had adrenal lesions identified on imaging, with the exception of two patients who had normal adrenal imaging.

All patients had biochemically confirmed PA based on the Endocrine Society guidelines [8]. Informed consent was obtained from all participants and AVS was performed without cosyntropin stimulation as described previously [9].

Serum samples were analysed for cortisol using the Abbott Alinity chemiluminescent microparticle immunoassay for cortisol with an analytical range 28–1650 nmol/L, precision, expressed as percentage coefficient of variation, of 7.2% at 100 nmol/L and 4% at higher concentrations (Abbott Laboratories, Maidenhead, UK). Aldosterone analysis was by an in‐house method for quantitation by liquid chromatography tandem mass spectrometry (LC‐MS/MS) following solid phase extraction with analytical range 60–5500 pmol/L, precision was below 6.6%. Plasma MN analysis used an in‐house LC‐MS/MS method following solid phase extraction, with analytical ranges for MN, NMN and 3‐methoxytyramine of 70−7093, 150−10,666 and 85−6585 pmol/L respectively. Precision was below 3.8% for all three metabolites. Samples above the analytical range were analysed following dilution with appropriate diluents.

The study focused on two primary outcomes: cannulation success and lateralisation, both treated as binary outcomes. Accurate interpretation of AVS depends on successful cannulation, which is confirmed by the SI. We calculated the SI using both cortisol (cortisol AV/cortisol PV) and MN (MN AV/MN PV). In our centre, a SI > 2 for cortisol (without tetracosactrin) indicates successful cannulation, as per the Endocrine Society Guidelines.

Lateralisation indices were defined as follows:

$${\mathrm{LI}}_{\mathrm{Cortisol}}=\;\frac{\frac{{\text{Aldosterone}}_{\text{dominant adrenal vein}}}{{\text{Cortisol}}_{\text{dominant adrenal vein}}}(\mathrm{AC})}{\frac{{\text{Aldosterone}}_{\text{non}-\text{dominant adrenal vein}}}{{\text{Cortisol}}_{\text{non}-\text{dominant adrenal vein}}}(\mathrm{AC})}$$

$${\mathrm{LI}}_{\mathrm{Metanephrine}}=\;\frac{\frac{{\text{Aldosterone}}_{\text{dominant adrenal vein}}}{{\text{Metanephrine}}_{\text{dominant adrenal vein}}}(\mathrm{AM})}{\frac{{\text{Aldosterone}}_{\text{non}-\text{dominant adrenal vein}}}{{\text{Metanephrine}}_{\text{non}-\text{dominant adrenal vein}}}(\mathrm{AM})}$$

The contralateral suppression index (CSI) was calculated as the AC in the non‐dominant AV divided by the AC in the PV. According to the Endocrine Society guidelines, unilateral disease is confirmed if the AC ratio of the dominant AV exceeds twice that of the non‐dominant AV. In our centre, we enhance confidence in identifying unilateral disease by ensuring that the CSI is below half of that of the IVC. The CSI cut off of <0.5 is based on our own retrospective analysis (unpublished data) and was 95% sensitive in identifying unilateral disease with specificity of 100%. This applies to our method without tetracosactrin stimulation and without sequential sampling of the AV and lower IVC [9].

The analysis of AM ratios in this study was conducted post hoc. Treatment decisions–whether medical or surgical–were based on AC ratios at the time, not AM ratios. To establish a cut‐off value for AM LI, we excluded patients with MACS or those without ONDST.

Six‐month clinical and biochemical outcomes data were evaluated using the PA Surgical Outcome (PASO) criteria [10]. Biochemical success was categorised as complete if hypokalaemia (when present) resolved and the aldosterone‐to‐renin ratio (ARR) normalised. Partial biochemical success was defined as correction of hypokalaemia with an improved but still elevated ARR, including *a* ≥ 50% decrease in aldosterone levels or improved confirmatory test results. Clinical success was deemed complete if blood pressure normalised without medication, partial if blood pressure improved with reduced medication and absent if there was no improvement or worsening despite treatment.

## 3 Statistical Analysis

Variables are presented as means ± standard deviation (SD) or medians and interquartile ranges for non‐normally distributed data. Receiver operating characteristic (ROC) curve analyses were performed to assess the diagnostic utility of plasma MN for predicting AV cannulation and lateralisation, using established AC ratios as reference criteria. The cut‐off point that optimised the combination of sensitivity and specificity was selected, with confidence intervals (CI) calculated using the exact binomial method. Concordance between plasma MN and cortisol results was evaluated using cross‐tabulation. Additionally, the Cohen kappa coefficient was used to measure the agreement in lateralisation between the AC and AM ratios. All analyses were conducted in RStudio (https://www.r-project.org/).

## 4 Results

The study included 129 patients, with a total of 131 procedures performed by a single operator. Two patients required a second procedure due to unsuccessful cannulation during the initial attempt. Patient characteristics and baseline data are summarised in Table 1.

**Table 1:** Baseline characteristics. Variables are presented as means ± SD or median and interquartile range for non‐normally distributed data.

*n* = 129	Baseline
Age (years)	50 ± 10
Gender Male	79 (61%)
Systolic Blood Pressure (mmHg)	164 ± 21
Diastolic Blood Pressure (mmHg)	98 ± 14
Body mass index (kg/m^2^)	26.2 ± 2.5
Serum potassium (mmol/L)	3.0 ± 0.49
Plasma renin activity (nmol/L/h)	0.31 ± 0.23
Aldosterone (pmol/L)	700 ± 530
Type 2 diabetes mellitus (%)	20 (16%)
Bilateral adenomas (%)	31 (24%)
Size of adrenal adenomas (mm)	12 ± 6.9
Number of antihypertensives	2 (1−3)
Mean cortisol on ONDST (nmol/L)	67 ± 50.3

Among the 129 patients, 72 underwent an ONDST to exclude or confirm MACS. Of these, 14 patients failed the test with post‐dexamethasone cortisol levels ranging from 55 to 210 nmol/L. In addition, these patients had low ACTH (<10 ng/L) and DHEAS levels, further supporting a diagnosis of MACS. Importantly, none of the patients had clinical features indicative of overt Cushing's syndrome.

### 4.1 Successful Cannulation

**Figure 1:**
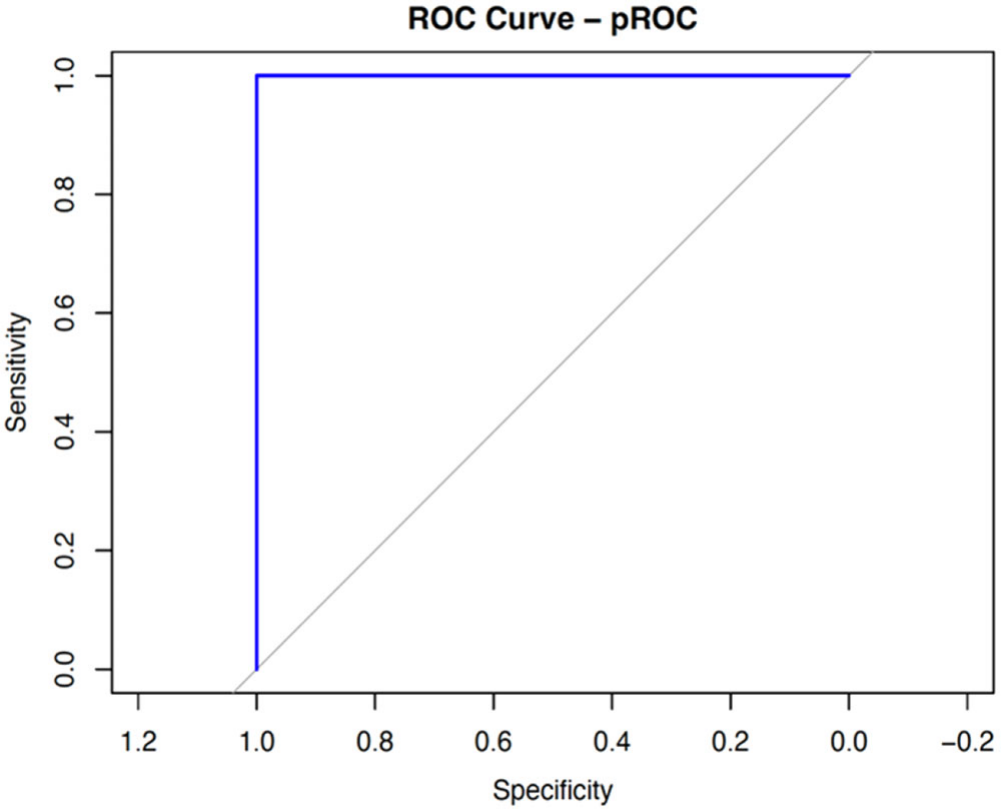
ROC curve illustrating the prediction of successful cannulation based on MN SI.

All of the 131 procedures had complete datasets and were included in the full analysis to determine the optimal cut‐off for MN SI using ROC analysis (Figure 1). We identified an optimal SI cut‐off value of >3, which achieved a sensitivity of 99% (95% CI, 95−99) and a specificity of 100% (95% CI, 40−100). The positive predictive value (PPV) was 100%, and the negative predictive value (NPV) was 80%. The area under the ROC curve (AUC) was 1.0, indicating excellent diagnostic accuracy. Further analyses evaluated the predictive ability of MN measurements using this cut‐off including a cross‐tabulation to assess cannulation success (Table 2).

**Table 2:** Cross‐tabulation of MN and cortisol results for successful cannulation.

*n* = 131	Successful cannulation (cortisol SI > 2)	
	No	Yes
MN SI < 3	4	1
MN SI > 3	0	126

Successful cannulation was confirmed in 126 patients using both MN and cortisol SIs. Four patients had failed cannulation according to both MN and cortisol SIs, while one patient failed based on MN but not on cortisol SIs (Table 2).

### 4.2 Lateralisation

**Figure 2:**
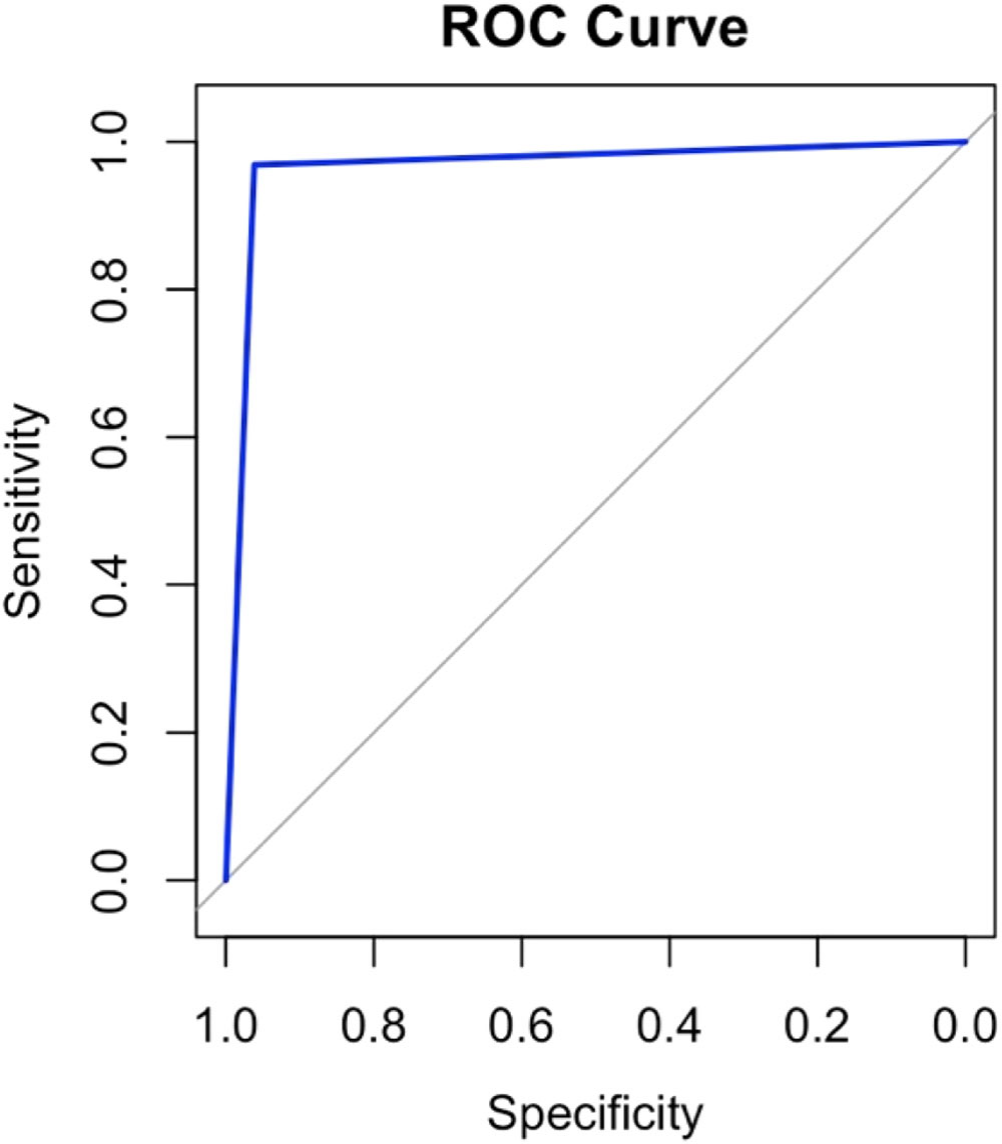
ROC curve for predicting unilateral disease using AM ratios.

To establish the MN LI, patients with MACS (*n* = 14) or without an ONDST (*n* = 54) were excluded, leaving 58 patients for analysis. ROC curve analysis (Figure 2) identified an optimal AM LI cut off of >4, yielding a sensitivity of 94% (95% CI, 81%−99%) and specificity of 96% (95% CI, 74%−98%). The PPV was 97%, the NPV was 93% and the overall accuracy was 94.8%. The AUC was 0.96, indicating excellent diagnostic performance. An AM LI > 4 suggests unilateral disease in patients who meet the criteria established for unilateral PA (AC LI > 2 and AC CSI < 0.5).

Three patients had discordant results. In two cases, the AM LI indicated unilateral disease, while AC LI suggested bilateral disease (Table 3); both patients are currently undergoing medical treatment. In the third case, the AM LI indicated bilateral disease, whereas the AC LI suggested unilateral disease; this patient subsequently underwent adrenalectomy.

**Table 3:** Cross‐tabulation of AM and AC results for lateralisation.

*n* = 58	Non‐lateralised AC LI < 2	Lateralised AC LI > 2	Total
AM LI < 4	25	1	26
AM LI > 4	2	30	32
Total	27	31	58

After establishing an AM LI cut‐off > 4 as an indicative of unilateral disease, this threshold was applied to the entire study cohort (Table 4). All 126 patients with successful cannulation were assessed for concordance between the AM and AC ratios. Of these, 69 had concordant results consistent with unilateral disease, while 50 had concordant results indicating bilateral disease, yielding a total of 119 concordant results (94.4%) (Table 4). The calculated Cohen's kappa coefficient was 0.89, demonstrating strong agreement between the AM and AC lateralisation indices.

**Table 4:** Application of AM LI > 4 to the entire study population.

*n* = 126	Non‐lateralised AC LI < 2	Lateralised AC LI > 2	Total
AM LI < 4	50	5	55
AM LI > 4	2	69	71
Total	52	74	126

Among the seven patients with discordant results, two had unilateral disease based on AM LI, while AC LI indicated bilateral disease. Both were non‐co‐secretors and are currently undergoing medical treatment. The remaining five patients were classified as having bilateral disease according to AM LI, with LIs ranging from 1.2 to 2.5, whereas the AC ratio suggested unilateral disease. Among these, three patients underwent adrenalectomy guided by the AC LI. Two were found to have unilateral adrenal adenomas, and one had bilateral adrenal adenomas on imaging, with histology confirming adrenal adenomas in all cases. The other two patients are currently awaiting surgery. Of those who underwent surgery, two achieved biochemical cure–one with full clinical success and the other with partial clinical improvement–while the third patient with bilateral adenomas did not achieve clinical or biochemical success.

Among patients with bilateral disease (*n* = 50), 5 had CSI values between 0.8 and 0.9. All five were found to have additional adrenal lesions on the side opposite to the AC lateralisation, consistent with radiologically bilateral disease. Based on the combination of AC ratios and borderline CSI values, a decision was made to pursue medical management rather surgical intervention.

### 4.3 MACS

Among the 14 co‐secretors (Table 5), AVS results were discordant in four patients with AC ratios suggesting bilateral disease and AM ratios indicating unilateral disease. Of the four co‐secretors with discordant results, three had unilateral adrenal adenomas identified on imaging, with AC LIs > 2; however, all three patients had a contralateral CSI > 1. One patient (Patient 6) was found to have bilateral adrenal nodules on imaging, an AC LI of 91.6 and a CSI of 0.9. Surgery was offered to all four patients based on their AC LI and imaging findings.

**Table 5:** AVS results in patients with MACS.

Patients	ONDST cortisol (nmol/L)	Laterality by AC ratios	AC LI	CSI	Laterality by AM ratios	AM ratio	Imaging	Treatment	Pathological Finding	Biochemical outcome	Clinical outcome
1	69	Left	23	0.4	Left	90.2 times more than right	15 mm left adrenal adenoma	left adrenalectomy	Adenoma	Unavailable	Unavailable
2	141	Bilateral	2.28	1.6	Right	8 times more than left	16 mm right adrenal adenoma	right adrenalectomy	Adenoma	Complete success	Complete success
3	149	Left	17	0.4	Left	500 times more than right	32 mm left adrenal adenoma	Left adrenalectomy	Adenoma	Complete success	Complete success
4	167	Bilateral	2.8	1.6	Bilateral	1.8 times more than right	13 mm left and 12 mm right adrenal nodules	Medical	—	—	—
5	82	Bilateral	16.6	1.25	Right	77.5 times more than left	23 mm right adrenal adenoma	Right adrenalectomy	Adenoma	Complete success	Complete success
6	171	Bilateral	91.6	0.9	Right	120 times more than left	21 mm left and 8 mm right adrenal nodules	Right adrenalectomy	Adenoma	Complete success	Complete success
7	54	Bilateral	1.38	1.98	Bilateral	1.23 times more than left	16 mm left and 12 mm right adrenal nodules	Medical	—	—	—
8	71	Bilateral	5.63	2.03	Left	9.8 times right	28 mm left adrenal adenoma	Left adrenalectomy	Adenoma	Complete success	Complete success
9	210	Bilateral	8	1.1	Bilateral	2 times more than right	24 mm left adrenal adenoma	Medical	—	—	—
10	53	Left	38.2	0.13	Left	94 times more than right	25 mm left adrenal adenoma	Left adrenalectomy	Adenoma	Complete success	Partial success
11	108	Bilateral	1.41	1.1	Bilateral	1.8 times more than right	13 mm left adrenal adenoma	Medical	—	—	—
12	107	Right	16.9	0.4	Right	42 times more than left	14 mm left and 26 mm right adrenal nodules	Right adrenalectomy	Adenoma	Partial success	Partial success
13	59	Right	72.9	0.12	Right	81 times more than left	25 mm right adrenal adenoma	Right adrenalectomy	Adenoma	Complete success	Complete success
14	55	Right	29.69	0.3	Right	48.8 times more than left	35 mm right adrenal adenoma	Right adrenalectomy	Adenoma	Unavailable	Complete success

*Note:* Four patients with MACS exhibited discordant results (Patients 2, 5, 6 and 8). Clinical and biochemical success was assessed using PASO criteria only, as no post‐operative ONDST was available.

Of the four patients with discordant results, four underwent unilateral adrenalectomy, with histological analysis confirming adrenocortical adenomas in all cases. All four patients achieved complete clinical and biochemical remission according to PASO criteria.

Two patients with bilateral adenomas on CT (patients 6 and 12) who were managed surgically do not have post‐operative ONDST available to confirm cure from MACS.

### 4.4 Adrenalectomy

Of the 76 patients who underwent adrenalectomy, 6‐month post‐operative clinical outcome data were available for 36 patients and post‐operative biochemical outcome data for 37 (Table 6). Among those classified as clinically successful, 21 achieved complete success, while 15 had partial success. Notable differences were observed between the groups regarding age (*p* = 0.017) and BMI (*p* = 0.0428). In terms of biochemical outcomes, 33 patients achieved complete success and four had partial success. Differences in BMI (*p* = 0.0051) and plasma renin activity (*p* = 0.008) were significant between the two groups, although other parameters did not show significant differences. Most patients had unilateral CT findings, and classical histopathology was similarly observed in those with complete clinical (76%) and biochemical (73%) cure at 6 months.

**Table 6:** Characteristics of patients who underwent surgery evaluated using the PASO criteria.

	Clinical (*n* = 36)			Biochemical (*n* = 37)		
	Complete success	Partial success	Overall *p* value	Complete success	Partial success	Overall *p* value
Outcomes	21	15	N/A	33	4	N/A
Age (years)	49 ± −8.9	57 ± 9.3	0.017	49 ± 10	58 ± −15	0.31
Gender						
Male	13 (62%)	8 (53%)		13 (40%)	3 (75%)	
Body mass index (kg/m^2^)	29.1 ± 5.3	32.4 ± 4.1	0.0428	28.9 ± 5.4	33.3 ± 3.4	0.0051
Plasma aldosterone (pmol/L)	743 ± 353	954 ± 815	0.37	845 ± 611	700 ± −201	0.34
Plasma renin activity (nmol/L/h)	0.31 ± 0.2	0.25 ± −0.12	0.27	0.28 ± 0.16	< 0.2	0.008
Serum potassium (mmol/L)	3.03 ± 0.40	3.24 ± −0.62	0.27	3.12 ± 0.53	3.1 ± −0.08	0.84
CT finding						
Unilateral	19 (90%)	12 (80%)		28 (85%)	0	
Bilateral	2 (10%)	3 (20%)		5 (15%)	4 (100%)	
Histopathology	16 (76%)	10 (67%)		24 (73%)	2 (50%)	
Classical Nonclassical	5 (24%)	5 (33%)		9 (27%)	2 (50%)	

## 5 Discussion

Accurate subtype diagnosis in PA is crucial for guiding appropriate management strategies. Unilateral aldosterone‐producing adenomas typically require surgical intervention, while bilateral disease is managed medically [11]. AVS remains the gold standard for subtype differentiation. Conventionally, cortisol has been used as the sole analyte to assess both the successful cannulation and the lateralisation of aldosterone secretion during AVS. However, cortisol has inherent limitations, including its prolonged half‐life and susceptibility to physiological fluctuations, which may affect its reliability as a marker in this context.

Some authors have proposed MN as a more precise alternative for determining selectivity in AVS procedures. Dekkers et al. reported that a MN SI > 12 indicates successful cannulation [12]. Carroll et al. found that a cortisol SI ≥ 5.0 aligns with a MN SI ≥ 12.0 for successful cannulation [13]. They also demonstrated that an AC ratio or AM ratio > 4 accurately determined lateralisation in 26 patients undergoing stimulated AVS, achieving a sensitivity of 100% and specificity of 94.1% with only one discordant result (PPV 91.6%, NPV 100%). The discordant case had an AM ratio that indicated unilateral disease, while AC ratio suggested bilateral disease. Imaging confirmed bilateral bulky adrenal glands, leading to decision for medical management.

Buffolo et al. identified eight unilateral PA cases that would have been misinterpreted by using the AC ratio, with three biochemically cured by surgery and five managed with medications [14]. Conversely, four patients diagnosed as unilateral by the AC ratio were reclassified as bilateral by the AM ratio; two were biochemically cured by surgery, while one declined surgery and one had persistent disease after surgery. The study lacked post‐adrenalectomy outcomes for some unilateral cases and did not screen for MACS.

In our study, we established hospital site‐specific cut‐off values for the MN SI and LI. A MN SI > 3 showed 96% agreement with cortisol SI. Additionally, an AM ratio > 4 demonstrated 94% concordance with the AC ratio. Our findings suggest that using both plasma MN and cortisol can enhance the robustness and confidence in interpreting AVS results. However, seven patients (6%) had discordant results between AC and AM ratios, with two patients on regular acetaminophen, which may affect plasma MN levels due to its known interaction with catecholamine metabolism. Three patients with discordant results underwent unilateral adrenalectomy based on the AC LI. Two achieved biochemical cure, both of whom had unilateral adenomas identified on imaging. In contrast, the patient with persistent PA post‐surgery was found to have bilateral adenomas, suggesting that AM LI was more accurate in predicting bilateral disease in this case. However, the sample size is too small to draw definitive conclusions about the superiority of AC LI over AM LI in patients without cortisol co‐secretion.

Plasma MN proved more reliable than cortisol in patients with MACS in cases with discordant results, when the AC LI suggested bilateral, but AM LI indicated unilateral disease. This emphasises the clinical importance of plasma MN in improving diagnostic accuracy and guiding treatment decisions, particularly in complex cases with cortisol co‐secretion, thereby minimising diagnostic errors and optimising treatment strategies. Importantly, no cases were found where AM lateralisation indicated bilateral disease while the AC ratio suggested unilateral disease in co‐secreting patients. Notably, none of the patients in our study required perioperative glucocorticoid therapy, and no case of post‐operative adrenal insufficiency was observed among those with MACS.

While current guidelines recommend to consider unilateral adrenalectomy after discussion by an expert multidisciplinary team in patients with MACS and associated comorbidities if a unilateral adrenal lesion is identified on imaging, concordance between CT and AVS appears to be just over 50% in various studies [15, 16, 17]. Guidelines acknowledge the limitations of CT in distinguishing small aldosterone‐producing adenomas from non‐functional nodularity. In addition, non‐functioning adrenal macroadenomas are a common finding in older people and small aldosterone producing microadenomas or bilateral adrenal hyperplasia may be missed with imaging‐guided PA subtyping alone. In our centre, current standard practice is to offer AVS to all patients with confirmed PA who are eligible and willing to undergo adrenalectomy.

A recent retrospective cohort study demonstrated that adrenalectomy, both unilateral and (partial) bilateral, can provide clinical and biochemical benefits for selected patients with bilateral PA [18]. At 6–12 months, 81% of unilateral and 92% of bilateral adrenalectomies showed clinical improvement, with higher biochemical success in bilateral diseases. Adrenal insufficiency occurred in 31% of bilateral diseases but was mostly transient.

In addition to the AM SI and LI findings, PASO data suggested that clinical and biochemical success rates were also influenced by factors such as age and in particular BMI in keeping with findings reported previously by Chidambaram et al. who showed that male gender and BMI were independent predictors of resolution of hypertension following unilateral adrenalectomy for PA [19]. Unilateral CT findings and classical histopathology were more common in patients who achieved complete success by PASO criteria. According to the international histopathology consensus for unilateral PA [20], classical histology is characterised by the presence of a solitary aldosterone‐producing adenoma, while non‐classical histology lacks a solitary APA or dominant aldosterone‐producing nodule, instead featuring multiple aldosterone‐producing micronodules, diffuse aldosterone‐producing hyperplasia, or no detectable aldosterone‐producing lesion. A recent study reported that patients with non‐classical histopathology had a higher rate of long‐term biochemical PA recurrence (60%) compared to those with classical histopathology (14%) [21]. While short‐term biochemical success was high for both groups, non‐classical histopathology is associated with a greater risk of recurrence over time.

The main limitations of this study include its retrospective design, with incomplete ONDST data across the cohort, potentially resulting in undiagnosed cortisol co‐secretion. As a tertiary referral centre, some patients were referred solely for AVS, and relevant clinical data from external institutions were not always available. Additionally, post‐adrenalectomy outcomes could not be assessed in all patients according to PASO criteria, and follow‐up ONDST data were unavailable for patients with MACS.

Despite its limitations, the study had several important strengths. The large sample size, the largest to date for analysing MN in unstimulated AVS, strengthens the reliability of the findings. Furthermore, screening outcome data for MACS in a large number of patients contributes to the study's robustness. A single operator performed all AVS procedures, reducing variability and ensuring a high rate of successful cannulation, which is crucial for accurate interpretation. A multidisciplinary review process for discordant AVS results ensured careful data interpretation and supported clinical decision making. Additionally, post‐adrenalectomy outcomes—such as improvements in blood pressure, plasma renin activity, aldosterone, serum potassium levels and histological confirmation of unilateral disease—further validated the accuracy of the LI.

In conclusion, while current guidelines recommend using the AC ratio for lateralisation, our findings indicate that incorporating the AM ratio enhances diagnostic accuracy and decreases the likelihood of missing unilateral disease, especially in patients with aldosterone‐cortisol‐co‐secreting adenomas. Integrating MN into conventional AVS analytes could assist clinicians in making more accurate treatment decisions and improve patient outcomes. Further research and validation across multiple centres will be essential to reinforce these recommendations and refine diagnostic protocols in AVS.

## Author Contributions

All authors contributed to the diagnosis and management of primary aldosteronism and were involved in the manuscript submission. Each author reviewed and approved the final version of the manuscript.

## Conflicts of Interest

The authors declare no conflicts of interest.
